# Internet Addiction Effect on Quality of Life: A Systematic Review and Meta-Analysis

**DOI:** 10.1155/2021/2556679

**Published:** 2021-12-06

**Authors:** Farzaneh Noroozi, Soheil Hassanipour, Fatemeh Eftekharian, Kumars Eisapareh, Mohammad Hossein Kaveh

**Affiliations:** ^1^Department of Health Promotion, School of Health, Shiraz University of Medical Sciences, Shiraz, Iran; ^2^Cardiovascular Diseases Research Center, Department of Cardiology, Heshmat Hospital, School of Medicine, Guilan University of Medical Sciences, Rasht, Iran; ^3^Institute of Health, Shiraz University of Medical Sciences, Shiraz, Iran; ^4^Research Center for Health Sciences, Institute of Health, Department of Health Promotion, School of Health, Shiraz University of Medical Sciences, Shiraz, Iran

## Abstract

**Purpose:**

Due to the use of different methodologies, tools, and measurements, the positive or negative impact of Internet use on human life quality is accompanied by a series of ambiguities and uncertainties. Therefore, in this study, a systematic review and meta-analysis are conducted regarding the effect of Internet addiction on the quality of life.

**Methods:**

A systematic search of resources was conducted to investigate the effect of Internet addiction on the quality of life. The databases of PubMed, Cochrane Library, Scopus, Web of Science, Embase, and Science Direct were searched from January 1980 to July 2020. The articles were screened by two researchers in multiple levels in terms of the title, abstract, and full-text; then, final studies that met the inclusion criteria were retrieved and included in the study.

**Results:**

After searching the previously mentioned international databases, 3863 papers were found, 18 of which we included in the final analysis. Surveys indicated that people who had a high Internet addiction received lower scores of quality of life than those who were normal Internet users (OR = 2.45, 95% CI; 2.31–2.61, *p* < 0.001; *I*^2^ = 85.23%, *p* < 0.001). Furthermore, There was a negative significant relationship between Internet addiction and quality of life in the psychological (OR = 0.56, 95% CI: 0.32–0.99, *p*=0.04, *I*^2^ = 97.47%, *p* < 0.001), physical (OR = 0.58, 95% CI: 0.39–0.86, *p*=0.007, *I*^2^ = 95.29%, *p*=0.001), and overall quality of life score (OR = 0.39, 95% CI: 0.27–0.55, *p* < 0.001, *I*^2^ = 0.0%, *p*=0.746).

**Conclusion:**

These findings illustrate that Internet addiction should be regarded as a major health concern and incorporated into health education and intervention initiatives.

## 1. Introduction

Among the different media types, the Internet is a recent achievement of mankind, a highly reachable global medium with an advanced modern communication technology capable of providing access to a wide range of information sources [1, 2].

Although the Internet and its technologies have provided valuable opportunities in scientific, communicative, and economic aspects for human societies, its inappropriate and extreme application, mostly for recreational purposes, is a serious threat to the health and well-being of the human population, especially young people [3]. According to studies, the increasing demand for Internet technology is associated with major health, psychological, and social problems, overwhelming mental health, interactions, and communications. Researchers also believe that excessive use of the Internet and social networks can indicate stress, anxiety, and depression; indeed, the excessive use of these networks is a way to reduce negative emotions [4].

The Internet affects various dimensions of lifestyle, social interaction, and occupational performance in both positive and negative ways. As to its positive effects, people can solve most of their daily problems via the Internet. In terms of developing interpersonal relationships, it goes beyond the geographical boundaries. Further, it has become an important part of everyday lives by helping exchange information and personal or professional experience, carry out economic/commercial activities, reduce transportation costs and problems, and develop business and marketing activities [5].

Negative effects have also been reported as real physical communications are decreasing compared with online communications due to the power of new technologies in the development and transformation of social communications, leading to weaker social relationships in the real world [6]. Overall, the Internet and social networks are not only changing human relationships and interactive patterns but also create intense interactions and influence individual life [7].

Internet addiction (IA) is an extreme form of this phenomenon, an inability to avoid using the Internet that has adverse effects on various life aspects (e.g., interpersonal relationships and physical health) [8]. It is considered a disorder in the Diagnostic and Statistical Manual of Mental Disorders, 5th Edition (DSM-5) [8, 9]. Estimation of the IA prevalence varies widely across countries (1.5% to 10.8%) [4, 10, 11]. Based on a meta-analysis result, its prevalence is 6% in 31 countries; the highest prevalence is 10.9% in the Middle East, and the lowest rate of 2.6% belongs to the north and west of Europe [8].

The studies of the IA show the reduction of life satisfaction in terms of family, friends, school, and living environment. References [12–14] have shown negative effects of IA on physical health aspects. As reported, the use of social networks causes insomnia, physical inactivity, and eye problems, as well as depression, social phobia, and hyperactivity disorders, in most users [12, 15]. Based on a meta-analysis by Ho et al., IA is significantly associated with alcohol abuse, attention deficit, hyperactivity, depression, and anxiety [16].

As mentioned, the Internet has a great impact on various aspects of health. According to the World Health Organization (WHO), health is defined as a complete state of physical, mental, and social well-being [17]. Quality of life (QOL) is a comprehensive measure of health outcomes [18]. It is a multidimensional concept that includes understanding mental and objective conditions of individuals' life in their sociocultural and economic environment [19]. The effect of excessive Internet use on health can be found by examining the impact of IA on QOL.

Several studies have indicated that IA reduces QOL [20]. On the contrary, some other studies have shown an insignificant association between the use of the Internet and social networks with QOL [21, 22]. For example, Ko et al. claim no relationship between QOL and moderate or intense Internet use [23]. A review study by Veenhoven, who examined IA and its derivatives, showed that the Internet increases QOL, and if the IA really exists, it can affect a relatively small percentage of an online population [24]. According to another review study, there is a positive relationship between using the Internet and computer and the QOL of the elderly [25]. Tran et al. have shown that the Internet can help people obtain a higher perceived QOL by promoting their work, education, and communication [26]. A cross-sectional study of college students found that the quality of life in daily users of social networking sites was higher than that of nondaily users [27].

On the other hand, a more in-depth study on types of applied programs on the Internet by individuals indicates the impact of a particular program on the individuals' mental well-being. In other words, spending time on programs involved with photo and video sharing is associated with higher levels of depression and anxiety; in contrast, using programs involved with book reading reduces depression and anxiety, thus increasing levels of mental well-being [28]. Researchers have also reported that people who spend much time online have lower perceived QOL due to the lack of long-term sleep, deteriorated physical health, difficulties in concentrating on work, and reduced intimacy with family members [29, 30].

The association between the Internet and the quality of human life is accompanied by a series of ambiguities and uncertainties due to the wide range of its potential positive and negative effects. Possible reasons may be different methodologies and tools, leading to differences in the measurement of Internet use rates. The selection of a specific and agreed form of inappropriate use, namely, the IA, as an independent variable and the definition and measurement based on well-known tools and standards of QOL as a consequence can probably result in precise findings on their relationship. According to the previously mentioned considerations, a systematic review and meta-analysis are conducted on the impact of IA on QOL.

## 2. Materials and Methods

The present study was a systematic review and meta-analysis. A systematic search of resources was conducted by a librarian (L.E) to investigate whether IA affects the QOL (condition) of people (population) across the world (context).

The research method was based on the PRISMA checklist [31].

### 2.1. Data Sources and Search Strategy

The Web of Science, Scopus, Cochrane Library, Embase, Science Direct, and PubMed databases from Jan 1980 to Jul 2020 were searched to find English articles. Also, SID and Magiran databases were searched for Persian studies. The grey literature and ongoing studies were searched in OpenGrey and Google Scholar; further, ProQuest was searched for thesis, dissertations, and studies presented at conferences.

The search was performed using MESH and free keywords. The keywords selected for the search were “Internet addiction” and “quality of life.” After determining relevant keywords, searches were done on databases using associated keywords with “AND” and “OR” operators combined together to determine relevant terms and synonyms. Search strategy included the following keywords: “compulsive Internet,” “computer addict,” “cyber addict,” “excessive Internet use,” “Internet addict,” “Internet dependent,” “Internet disorder,” “Net addict,” “online addict,” “quality of life,” “life quality,” and “health related quality of life.” The PubMed advanced mesh search features used for example were: (((((((((((((“quality of life” [MeSH Terms]) OR (“value of life” [Title/Abstract])) AND (impact [Title/Abstract])) AND (“Internet addiction” [Title/Abstract])) OR (“problematic Internet use” [Title/Abstract]) OR (“online gaming addiction” [Title/Abstract])) OR (“game addiction” [Title/Abstract])) OR (“excessive Internet use” [Title/Abstract])) OR (“social media addiction” [Title/Abstract])) OR (“Internet dependency” [Title/Abstract])) OR (“pathological Internet use” [Title/Abstract])) OR (“computer addiction” [Title/Abstract])) OR (“social networking addiction” [Title/Abstract])) OR (“pornography addiction” [Title/Abstract])). The complete search strategy of other databases is in Supplementary File 1.

The collected information entered EndNote, X7 (Thomson Reuters, Carlsbad, CA, USA), and duplicate papers were automatically deleted.

All cross-sectional, case-control, and cohort studies that examined the relationship between IA and QOL were searched.

### 2.2. Inclusion Criteria

- The study type had to be observational (cross-sectional, case-control, and cohort).
- The study was required to investigate the relation between IA and quality of life.
- The correlation level (*r*) between IA and quality of life had to be presented, or information based on which the correlation could be computed was required to be given.
- Papers had to be in English (due to the lack of translators for other languages) and Persian.

### 2.3. Exclusion Criteria

- The authors did not provide further information upon request, including the correlation level (*r*) between IA and QOL.
- Articles that had full texts written in non-English or non-Persian in spite of having abstracts in English or Persian were excluded.
- The study type was nonobservational (qualitative and interventional studies).

### 2.4. Study Selection

The selected articles were screened in multiple levels based on the title, abstract, and full-text; then, final studies that met the inclusion criteria were retrieved and included in the study. The initial search was conducted by two people. If there was unmatching between them, the team's supervisor (corresponding author) announced the final comment on that paper.

### 2.5. Articles' Quality Assessment

The STROBE checklist was used to check and control the quality of papers. This tool consists of 22 questions classified into “yes, no, and unclear.” It aims to assess the methodological quality of studies and strategies to identify bias in designs, implementations, and analyses in studies. During the evaluation process, papers with less than 50% of the inclusion criteria were excluded from the study [32].

### 2.6. Data Extraction and Quality Assessment

The information extracted from the articles was entered in the extraction form. Extracted data included: first author, year of publication, study name, country of study, sample size, sample characterization, age mean (SD), and study instrument.

### 2.7. Statistical Analysis

The heterogeneity between studies was examined by Cochran's test (with a significant level less than 0.1) and its composition using *I*^2^ statistics (with a value greater than 50%). A random-effect model was used in the presence of heterogeneity, while a fixed-effect model was used in its absence. The odds ratio (OR) index, obtained from the comprehensive meta-analysis (CMA) software was used for comparing meta-analysis results. All analyses were done using the statistical CMA 2 software.

## 3. Results

### 3.1. Search Results

**Figure 1:**
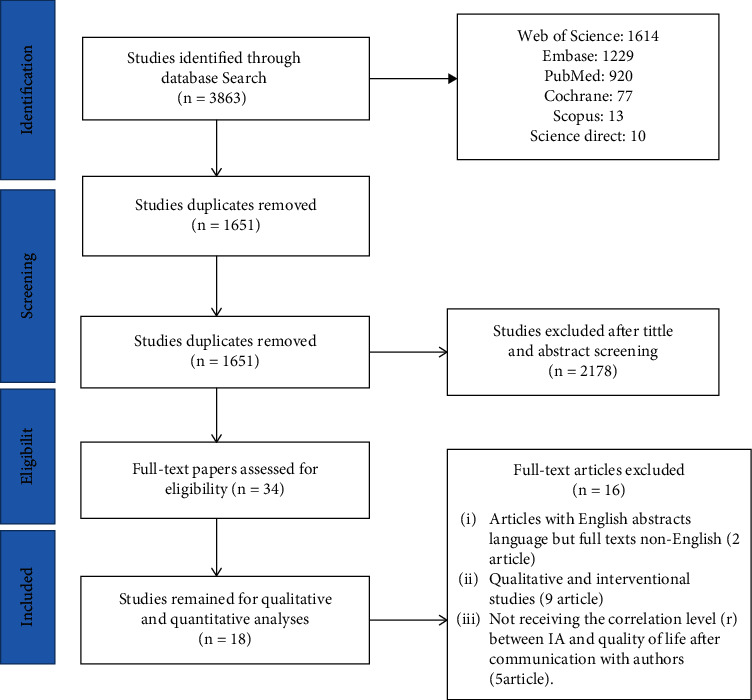
Flowchart of the included studies in systematic review.

Studies were reviewed and selected in three stages. At the first stage, 3863 papers from bases using keywords were retrieved and transferred to the reference management software (Endnote). Titles of papers were reviewed, and 1651 repetitive and 2178 irrelevant papers (to the main subject of research) were deleted. At the second stage, 34 papers associated with the main purpose of the project were selected by studying 2212 abstracts of the remaining papers. At the third stage, 14 studies were included in the final review by investigating the full text of 34 papers and considering inclusion criteria. The papers excluded at this stage were those with English abstract but non-English full text (two articles) and qualitative and interventional methodologies (9 articles), and not receiving the correlation level (*r*) between IA and QOL after communicating with authors (five articles). Finally, the results were evaluated using 18 papers eligible for inclusion in the study.  Figure 1 shows the process of retrieving and selecting articles.

### 3.2. Articles' Quality Assessment

All studies met more than 50% of the inclusion criteria (medium or high quality) and no studies were excluded during the evaluation process.

### 3.3. Characteristics of Included Studies

Table 1 presents the specifications of the articles investigated [33].

### 3.4. Statistical Analysis

The meta-analysis results were divided into several sections in the present study: first, the comparison of QOL of ordinary people with IA people based on overall scores of QOL and each of its dimensions; second, the analysis of the relationship between the severity of IA and QOL based on *r* index and calculated OR index.

Due to the high heterogeneity of the analysis, the relationship between the severity of Internet addiction and each dimension of the quality of life, a power analysis was performed. The high power of the analysis for each dimension of the quality of life showed that the results of the study were not affected by heterogeneity.

### 3.5. Comparing the Quality of Life of Ordinary People with Internet Addicts

Four studies examined the overall scores of both groups. Based on the results, people with a high IA (779 participants) received lower scores of QOL than those with normal Internet use (2589 participants) (95% CI: 2.31–2.61; *I*^2^ = 85.23%, *p* < 0.001).

**Figure 2:**
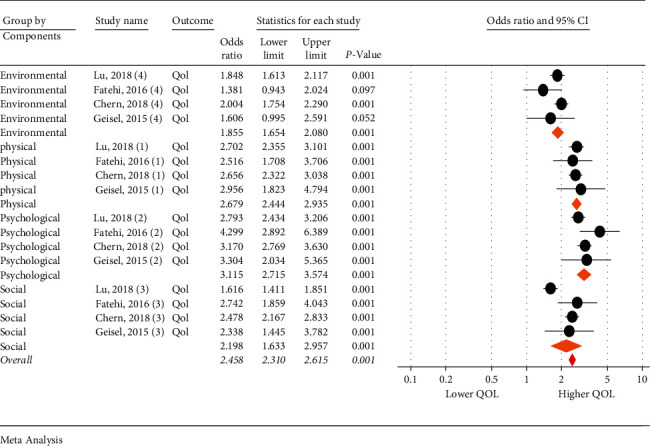
Comparing the quality of life of ordinary people with that of internet addicts.

Four studies examined other QOL dimensions. Based on the obtained results, people with severe IA received lower QOL scores than those with normal Internet use in terms of the environmental (95% CI: 1.65–2.08; *I*^2^ = 22.45%, *p*=0.276), physical (95% CI: 2.44–2.93; *I*^2^ = 0.0%, *p*=0.962), psychological (95% CI: 2.71–3.57; *I*^2^ = 38.32%, *p*=0.182), and social dimensions (95% CI: 1.63–2.95; *I*^2^ = 86.31%, *p* < 0.001).  Figure 2 shows results of the Forest plot for comparison of the QOL of ordinary people with IA (Figure 2).

### 3.6. The Relationship between the Severity of Internet Addiction and Quality of Life

**Figure 3:**
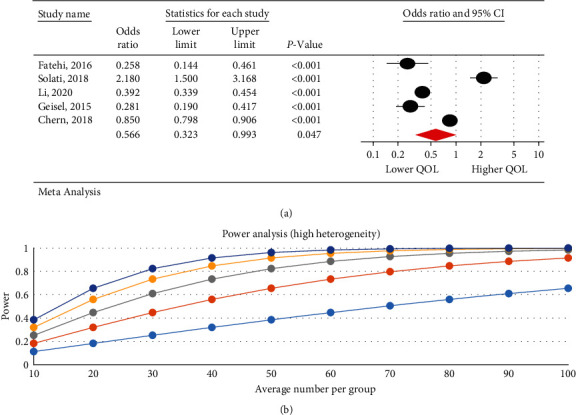
The relationship between the severity of internet addiction and the quality of life in the psychological dimension.

**Figure 4:**
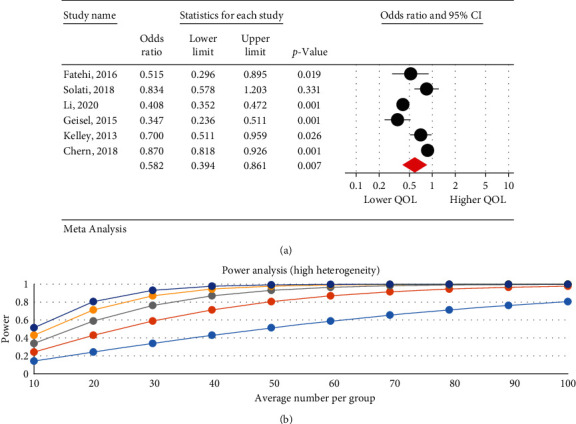
The relationship between the severity of internet addiction and the quality of life in the physical dimension.

**Figure 5:**
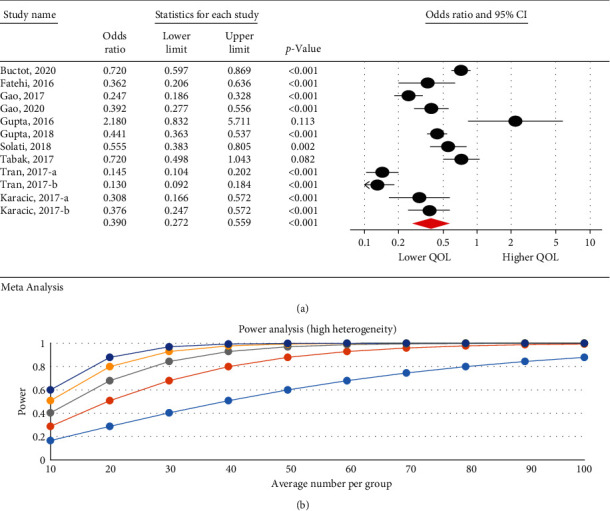
The relationship between the severity of internet addiction and the overall quality of life score.

**Figure 6:**
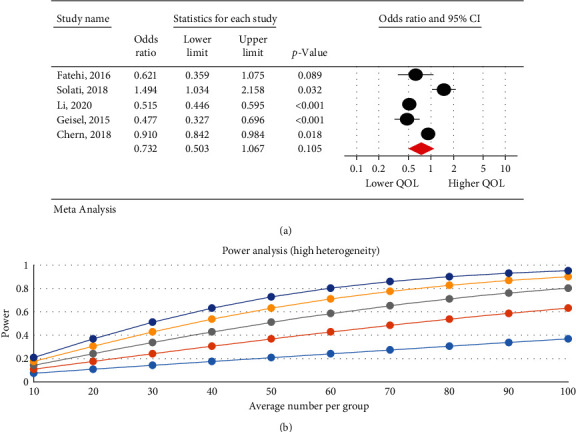
The relationship between the severity of internet addiction and the quality of life in the environmental dimension.

**Figure 7:**
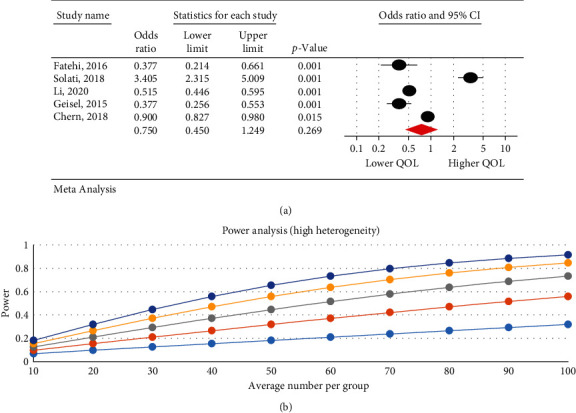
The relationship between the severity of internet addiction and the quality of life in the social dimension.

The research results indicated that IA is associated with a decrease in QOL. There was a negative significant relationship between the severity of IA and QOL in psychological (95% CI: 0.32–0.99; *I*^2^ = 97.47%, *p* < 0.001) (Figure 3), physical (95% CI: 0.39–0.86; *I*^2^ = 95.29%, *p*=0.001) (Figure 4), and overall QOL (95% CI: 0.27–0.55; *I*^2^ = 92.7%, *p* < 0.001) (Figure 5); however, no statistical significant reduction was observed in environmental (95% CI: 0.50–1.06; *I*^2^ = 93.89%, *p* < 0.001) (Figure 6) and social dimension (95% CI: 0.45–1.24; *I*^2^ = 96.63%, *p* < 0.001) (Figure 7).

**Figure 8:**
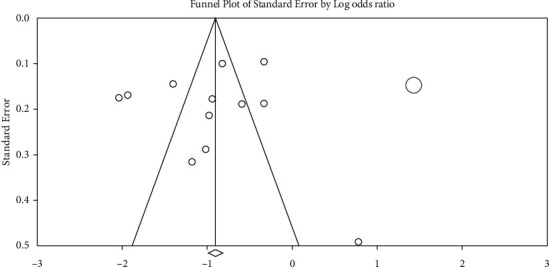
Funnel plot for assessing possible publication bias.

Based on the results of the Egger (*p*=(0/601)) and Begg test (*p*=(0/945)), no publication bias was observed among studies due to the symmetry of the funnel plot (Figure 8).

## 4. Discussion

Despite the increasing volume of research on the relationship between IA and QOL, no systematic review or meta-analysis has been conducted to summarize the findings to the best of the authors' knowledge. More specifically, the first study assessing IA and QOL has been published in 2013 [34]. Accordingly, the association between IA and sleep has been studied over the last seven years, and the cumulative evidence requires to be summarized. The present review is the first meta-analysis that uses empirical evidence from the past seven years to understand the association between IA and QOL. By a rigorous selection method using PRISMA guidelines, 18 studies with 11,097 participants were included in the present meta-analysis.

The high power of analysis for each dimension of quality of life showed that the results of the study were not affected by heterogeneity; one of the reasons could be the high number of samples in the study.

Meta-analysis results show differences in QOL based on Internet usage. As the results of four studies show that people with a high IA receive lower scores of QOL than those with normal Internet use (OR: 2.45, *p* < 0.001). This result was consistent with those of other studies in the field [35–38]; these results suggest that, in comparative studies, even after controlling some background variables affecting QOL, there are still significant independent correlations between IA and all aspects of QOL.

According to the obtained results of the meta-analysis (11studies), IA is associated with a decline in overall QOL (OR: 0.39; *p* < 0.001). This result, except in one [39] case, is consistent with the results of other studies included in the analysis [36, 40–48]. In these studies, an Indian study had the smallest sample size which was 60 [39], and a Filipino study had the largest sample size, which was 1447 [40]. The studies are also conducted across 11 countries mostly located in Asia (*n* = 11), followed by Europe (*n* = 5) and the USA (*n* = 2). Although the meta-analysis results in the present review are primarily derived from Asian populations, based on the Egger (*t*: 0.539, *p* : 0.601) and Begg test (*z*: 0.137, *p* : 0.190), no publication bias is observed among them. Additionally, with moderate- and high-quality studies using the STROBE checklist and standard measurement tools, the methodological concerns might have minimal impacts on the present findings.

The research results indicate a significant negative relationship between IA and QOL in the psychological (OR = 0.56, *p*=0.04) and physical dimensions (OR = 0.58, *p*=0.007). Different and sometimes contradictory results are reported in studies on the impact of IA on the QOL dimensions. For instance, in two studies by Solati et al. in Iran [44] and Kelley and Gruber in USA [34], IA decreased the QOL physical effect. Further, in a study by Lu et al. in China, IA reduced QOL in terms of physical, psychological, and environmental aspects [35]. Fatehi et al. [36] in Iran showed that IA decreased the QOL in physical, psychological, and social dimensions [36]. The results of three studies in Taiwan [37], China [49], and the USA [38] indicated that IA decreased the QOL in physical, social, psychological, and environmental aspects. In addition to a small number of cross-sectional studies, which make the comparison and deduction of causal relationships difficult, differences in contexts and ignorance of the underlying factors affecting the QOL dimensions (such as unemployment, chronic diseases, mental/psychological disorders (depression, negative feelings, and stress)) can be considered as reasons for the contradiction between results on the Internet impact on QOL dimensions [45, 50, 51].

On the other hand, a study conducted in Taiwan shows three specific IA manifestations (compulsive, interpersonal, health, and time management problems) to reduce the physical dimensions of QOL among college students. A possible explanation is that participants with higher compulsivity may have impaired control over Internet use, thereby developing the other two types of IA problems manifested through unhealthy lifestyles, such as poor diet and sleep deprivation, leading to lower physical QOL. Also, compulsivity concerning Internet use may cause poor mental health (depression, loneliness, anxiety, and stress), harming psychological HRQOL [37].

A longitudinal study in Hong Kong show that time management problem (staying online longer than originally intended) is considered the most common among the participants during the study period [52]. Such findings show the need for the implementation of IA intervention programs (time management, self-regulation, and self-efficacy) to prevent the deterioration of IA-related physical HRQOL.

### 4.1. Strengths and Limitations

Despite the increasing influence of the Internet in daily life, in the last eight years, no meta-analysis study has been conducted to investigate the effect of IA on QOL, and this study is the first study in this period.

The quality of the studies has been determined according to the information in the articles, and it is possible that the studies were of higher quality but did not provide all the information and as a result were in the group of medium-quality articles.

The study protocol was not registered before the start for this review and is considered as one of the limitations of the study because there is a concern that it may add to the possible bias over time.

## 5. Conclusion

According to the present review results, the Internet negatively affects overall QOL, physically and psychologically. Since the Internet meets the needs of information, entertainment, and social interactions, its use is an integral part of everyday human life (both work and leisure). Internet use can also trigger a compulsive need in a minority of individuals. These findings show that IA should be regarded as a major health concern and incorporated into health education and intervention initiatives. Also, further studies are suggested, in particular with a cohort and empirical design in different societies, using standardized methodologies and analytical reports that facilitate the comparison.

## Figures and Tables

**Table 1:** Data extraction results from studies.

Author/year	Country	Study population	Age mean (SD)	Sample size	QOL instrument	IA instrument
Fatehi et al. (2016)	Iran	7–4-year medical students	22.57 ± 1.2	174	WHOQOL-BREF	IAT
Li et al. (2018)	China	High school students	15.1 ± 1.9	1385	WHOQOL-BREF	IAT
Chern et al. (2018)	Taiwan	Students	20.51 ± 1.8	1452	HRQOL	IAT
Gupta et al. (2016)	India	Adolescent (18–23 years)	—	60	WHOQOL-BREF	IAT
Geisel et al. (2015)	USA, UK, Canada	Adult social network gamers	38:9 ± 13.4	370	WHOQOL-BREF	IAT
Kamal Solati (2018)	Iran	Students of Islamic Azad university	—	381	WHOQOL-BREF	IAT
Li et al. (2020)	China	University students	20.3 ± 1.6	2312	WHOQOL-BREF	The mobile phone addiction scale (MPAS)
Kelley and Gruber (2013)	USA	Undergraduate students (18 to 39 years old)	19.6 ± 2.96	133	SF-36v2 health survey	Problematic internet use questionnaire (PIUQ)
Gupta et al. (2018)	India	Adolescent (18–23 years old)	—	23	WHOQOL-BREF	IAT
Gao et al. (2020)	Germany	College students and highly educated adults.	25.8 ± 11.6	446	WHOQOL	ISS-10 (short version of the ISS-20)
Tabak and Zawadzka (2017)	Polish	Students	16.04 ± 0.9	376	KIDSCREEN-10 index	YDQ (8 items)
Tran et al. (2017)	Vietnamese	Young (15–25 years old)	21.5 ± 3.8	566	EuroQol	IAT
Tran et al. (2017)	Vietnamese	young (15–25 years old)	21.7 ± 1.7	586	EuroQol	IAT
Buctot et al. (2020)	Filipino	Adolescents (13–18 years old)	15.22 ± 1.61	1447	KIDSCREEN-27	Smartphone addiction scale short version (SAS-SV)
Gao et al. (2017)	Chine	University students	20.50 ± 1.4	722	WHOQOL-BREF	Mobile phone addiction scale (MPAS)
Paolo Soraci et al. (2020)	Italian	Online survey via Google forms	33.8 ± 16.2 18–99 years	205	Quality of life measure	Smartphone application based addiction scale (SABAS)
Karacic et al. (2017)	Germany	Students of primary and high school	11–18 years	149	SF-36	I IAT
Silvana Karacic et al. (2017)	Croatian	Students of primary and high school	11–18 years	310	SF-36	I IAT

## Data Availability

The data used to support the study are available from the corresponding author upon request.

## Abbreviations

IA: Internet addiction
QOL: Quality of life
OR: Odds ratio
HRQOL: Health-related quality of life
PRISMA: Preferred reporting items for systematic reviews and meta-analysis
WHO: World Health Organization.
