# Supplementary material for: Internet Addiction Effect on Quality of Life: A Systematic Review and Meta-Analysis
Source: ScientificWorldJournal. 2021 Dec 6;2021:2556679. doi: 10.1155/2021/2556679 (PMC8668296; doi:10.1155/2021/2556679)
Supplement: Supplementary Materials — The complete search strategy of other databases is provided as Supplementary File 1. [file 2556679.f1.doc]

**Search strategy:**

**PubMed = 920** results

**((((((((((((("quality of life"[MeSH Terms]) OR ("value of life"[Title/Abstract])) AND (impact[Title/Abstract])) AND ("internet addiction"[Title/Abstract])) OR ("problematic internet use"[Title/Abstract]) OR ("online gaming addiction"[Title/Abstract])) OR ("game addiction"[Title/Abstract])) OR ("excessive internet use"[Title/Abstract])) OR ("social media addiction"[Title/Abstract])) OR ("internet dependency"[Title/Abstract])) OR ("pathological internet use"[Title/Abstract])) OR ("computer addiction"[Title/Abstract])) OR ("social networking addiction"[Title/Abstract])) OR ("pornography addiction"[Title/Abstract]))**

**Scopus = 13** results

[( ( TITLE-ABS ( "quality of life" )  OR  TITLE-ABS ( "value off life" )  AND  TITLE-ABS ( impact ) ) )  AND  TITLE-ABS ( "internet addiction" )  OR  TITLE-ABS ( "problematic internet use" )  OR  TITLE-ABS ( "online gaming addiction" )  OR  TITLE-ABS ( "game addiction" )  OR  TITLE-ABS ( "excessive internet use" )  OR  TITLE-ABS ( "social media addiction" )  OR  TITLE-ABS ( "internet dependency" )  OR  TITLE-ABS ( "pathological internet use" )  OR  TITLE-ABS ( "computer addiction" )  OR  TITLE-ABS ( "social networking addiction" )  OR  TITLE-ABS ( "pornography addiction" )](https://www.scopus.com/results/documentSpellSuggest.uri?sort=plf-f&src=s&sid=fb354aa5f5d99973dc719b85d7480392&sot=a&sdt=a&sl=553&s=(+(+TITLE-ABS+(+"quality+off+life"+)+OR+TITLE-ABS+(+"value+off+life"+)+AND+TITLE-ABS+(+impact+)+)+)+AND+TITLE-ABS+(+"internet+addiction"+)+OR+TITLE-ABS+(+"problematic+internet+use"+)+OR+TITLE-ABS+(+"online+gaming+addiction"+)+OR+TITLE-ABS+(+"game+addiction"+)+OR+TITLE-ABS+(+"excessive+internet+use"+)+OR+TITLE-ABS+(+"social+media+addiction"+)+OR+TITLE-ABS+(+"internet+dependency"+)+OR+TITLE-ABS+(+"pathological+internet+use"+)+OR+TITLE-ABS+(+"computer+addiction"+)+OR+TITLE-ABS+(+"social+networking+addiction"+)+OR+TITLE-ABS+(+"pornography+addiction"+)&origin=resultslist)

**Embase = 1229** results

('quality of life':ab,ti OR 'value off life':ab,ti) AND impact:ab,ti AND 'internet addiction':ab,ti OR 'problematic internet use':ab,ti OR 'online gaming addiction':ab,ti OR 'game addiction':ab,ti OR 'excessive internet use':ab,ti OR 'social media addiction':ab,ti OR 'internet dependency':ab,ti OR 'pathological internet use':ab,ti OR 'computer addiction':ab,ti OR 'social networking addiction':ab,ti OR 'pornography addiction':ab,ti

Science direct: 10 results

(Impact AND quality of life) AND (internet addiction OR internet dependency OR computer addiction OR social networking addiction OR problematic internet use OR excessive internet use OR cyberspace addiction)

Wos: 1614 result

(TS= (internet AND dependence) OR TS= (problematic AND internet AND use) OR TS= (internet AND use) OR TS= (computer AND addiction) OR TS= (social AND networking AND addiction) OR TS= (internet AND dependency) OR TS= (cyber AND relationship AND dependency) OR TS=symptomatology) AND TS=impact AND TS=quality of life

Cochrane: 77 results

 Trials matching "internet addiction" in Title Abstract Keyword OR "problematic internet use" in Title Abstract Keyword AND "social media addiction" in Title Abstract Keyword AND "Quality of Life" in Title Abstract Keyword AND impact in Title Abstract Keyword - (Word variations have been searched)
